# Radiographic and computed tomographic evaluation of supinator sesamoid bones in dogs

**DOI:** 10.1186/s12917-022-03450-x

**Published:** 2022-10-24

**Authors:** Yasamin Vali, Aquilino Villamonte-Chevalier, Manon Dorny, Eberhard Ludewig, Henri van Bree, Ingrid Gielen

**Affiliations:** 1 Diagnostic Imaging, Department of Companion Animals and Horses, University of Veterinary Medicine Vienna (Vetmeduni), Vienna, Austria, Veterinaerplatz 1, 1210 Vienna, Austria; 2 Department of Veterinary Medical Imaging and Small Animal Orthopaedics, Ghent University, Merelbeke, Ghent, Belgium, Salisburylaan 133, 9800 Merelbeke, Belgium; 3 VetMedImage, Hoogstraat 48, 9420 Erondegem, Belgium

**Keywords:** Sesamoid bone, Supinator muscle, Computed tomography, Radiography, Canine, Elbow dysplasia

## Abstract

**Background:**

The present study evaluated the frequency of supinator sesamoid bones (SSB) on radiography and computed tomography (CT). Interobserver agreement was evaluated in the detection of the SSBs in both methods. A correlation between the existence of SSBs and elbow diseases (ED) was assessed. For these purposes, radiographs, and CT scans of 100 dogs were scored by 3 observers.

**Results:**

The SSB was identified as a round to oval-shaped opacity and measured 0.5–6.56 mm × 0.5–6.2 mm. SSBs were reported in an average of 8,33% of dogs on radiographs and 26% of dogs on CT; a bilateral sesamoid bone was present in 43,52% and 76,92% of these dogs, respectively. Seventy-two percent of the SSBs was identified on CT were not detected on radiographs. The Kappa test showed a substantial agreement (κ = 0.691) and a perfect agreement (κ = 1) between the observers in the detection of SSBs on radiography and on CT scans respectively. Additionally, a weak positive correlation was detected between ED and the existence of SSBs.

**Conclusion:**

A supinator sesamoid bone can be detected occasionally in the evaluation of the canine elbow joints by routine radiography. CT is superior to radiography for assessing SSBs with a higher interobserver agreement. The correlation of the existence of the SSBs and ED, needs further evaluations to prove a probable pathophysiological connection.

## Background

The supinator muscle is a broad and flat muscle which lies laterally on the elbow directly on the joint capsule and radius [1]. It has a thick tendon which arises on the lateral collateral ligament of the elbow joint and lies on the lateral epicondyle [1]. This muscle is responsible for the supination of the paw, like its name suggests, so that the palmar surface faces medially [1]. A sesamoid bone can be found in the tendon of the supinator muscle’s origin which articulates with the craniolateral aspect of the head of the radius and it is tightly connected to the joint capsule [2]. The supinator sesamoid bone (SSB) is described in the elbow joint of cats [2] and larger breed dogs [3]. It has a distinct border, and its shape varies from circular to irregular oval. Physiologically sesamoid bones develop within a soft tissue structure such as a tendon or joint capsules where they contact a bony structure, but they can also be formed prenatally [1]. According to Evans and De Lahunta (2013), sesamoid bones have 3 important functions: protect tendons from bony prominences, increase surface for attachment of tendons and change the direction of pull [1]. Thus, hypothetically the sesamoid bones can be formed in response to repeated stress and excessive mechanical friction occurring in a tendon, and it is possible that the presence of SSB also may be associated with elbow pathologies which change the normal tension of the adjacent structures. On the other hand, the detection of sesamoid bone in the supinator muscle tendon is also of relevance since it can be mistaken for other pathologies and fragmentation of the bony structures. It is important to know that not all sesamoid bones can be identified on radiographs, as sometimes they are still cartilaginous at the time of radiography and in some dogs SSBs are unilaterally present or absent [4]. It is noticeable that the term “Supinator Sesamoid Bone- SSB” used in the present study refers to the mineralized SSBs.

Based on the present knowledge, the objectives of this study are:

1. To evaluate the frequency of the presence of the sesamoid bone on radiographs and on CT images,
2. To assess the detection of the SSBs in radiography and CT by means of an interobserver agreement,
3. To evaluate the correlation of the elbow disease and presence of the SSB.

## Material and methods

This study was a retrospective design, so no institutional animal care and use approvals were requested officially and a written informed consent was obtained from the owners for the participation of their animals potentially in retrospective studies at the time of admission in the hospital.

The database of the Department of Veterinary Medical Imaging and Small Animal Orthopaedics of Ghent University, Ghent, Belgium was searched from 2007 to 2018 to select 50 dogs with, and 50 dogs without an elbow disease. Cases were included if they had radiographic and CT examination of their both elbow joints. All the cases were assessed and scored by 2 experienced radiologists and 1 non-experienced veterinary student. The guideline was presented to the observers to record the presence of a sesamoid bone, arthrosis, elbow diseases (incomplete ossification of the humerus condyles, elbow dysplasia: ununited anconeal process, incongruity, medial coronoid process disease, osteochondrosis) and to measure the dimensions of the sesamoid bone. The studies were anonymized by analyzed using work-station software Osirix (Osirix Medical Imaging Software version 4.1.2 DICOM viewer, Pixmeo, Bernex, Switzerland).

Based on the protocol of the department of Veterinary Medical Imaging and Small Animal Orthopaedics of Ghent University for radiography of elbow joints, all the dogs were sedated using dexmedetomidine (0.005 mg/kg of body weight, Dexdomitor, Finland). Three standard radiographic views were taken of each elbow joint, a mediolateral extension, a mediolateral flexion and a craniolateral-caudomedial oblique (Cr15°LCdMO) radiographs using 55 kVp and 8 mAs.

The radiographs were taken using a digital radiography system (EKLIN EDR6, Canon medical systems, Netherlands). For the mediolateral projections, dogs were positioned in lateral recumbency. The upper limb was pulled caudally and the head was pulled back to prevent superposition. Ideally, the elbows on the dependent side were positioned in 120° for the extended view and less than 45° for the flexed view. The X-ray beam was centered on the medial epicondyle. For the craniolateral-caudomedial oblique (Cr15°LCdMO) view the dogs were placed in the sternal position with the for limbs extended cranially and the limb was pronated 15°. The head was pulled back to prevent superposition. The X-ray beam was centered on the joint-space distal to the medial epicondyle of the humerus. Both legs were X-rayed in this position.

Immediately after radiography, the dogs were anesthetized using an IV bolus of 2 mg/kgBW propofol (Propovet, Schering-Plough, Comazzo, Italy) and intubated. Anesthesia was maintained with isoflurane (IsoFlo, Abbott Laboratories, Queensborough, UK) in 100% oxygen. The dogs were positioned in left lateral decubitus with the forelimbs extended cranially. The head of the dogs was pulled back laterally to prevent superposition and artifacts. CT scans were performed by a four-slice scanner (Lightspeed Qx/I, General Electric Medical Systems, Milwaukee, WI) using 120 kVp, 140 mA and 25 cm field of view, 1.3 mm thickness from the proximal aspect of the olecranon to 2 cm distal of the elbow joint using a bone algorithm (De Rycke et al., 2002).

The Chi square (χ2) test was used with a 2 × 2 contingency table to check the correlation between the occurrence of SSBs and occurrence of elbow disease. This test was performed with and without the Yates correction. Also, the Phi and the Cramer’s V value were calculated. The agreement between the observers in identification of the SBBs in both radiographs and CT images were evaluated by Cohen’s kappa test. The result of the Cohen’s Kappa test was interpreted based on the guideline presented by McHugh 2012 [5]. The value of Kappa between 0 and 0.20 was interpreted as “no agreement”, 0.21–0.39 as “minimal agreement”, 0.4–0.59 as “weak agreement”, 0.60–0.79 as “moderate agreement”, 0.80–0.90 as “strong agreement” and above 0.90 as “almost perfect agreement” (McHugh 2012). Statistical analysis was performed using SPSS (version 19.0; IBM, Chicago, USA).

## Results

A total of 100 dogs (200 elbow joints) were included in the present study. The included dogs had a mean age of 20 months (range: 4 to 92 months), 60 dogs were male, and 40 dogs were female. The included breeds were Golden Retrievers (*n* = 42), Labrador Retrievers (*n* = 34), Cavalier King Charles Spaniel (*n* = 4), Bernese Mountain Dog (*n* = 3), German Shepherd (*n* = 2), Malinois (*n* = 2), White Swiss Shepherd (*n* = 1), Cane Corso (*n* = 1), American Staffordshire Terrier (*n* = 1), Bordeaux dog (*n* = 1), Boxer (*n* = 1), Border Collie (*n* = 1), Australian Shepherd (*n* = 1), English Springer Spaniel (*n* = 1), English Cocker Spaniel (*n* = 1), Labione (*n* = 1), Stabyhound (*n* = 1), Brittany Dog (*n* = 1) and the breed was not recorded in 1 dog.

**Fig. 1:**
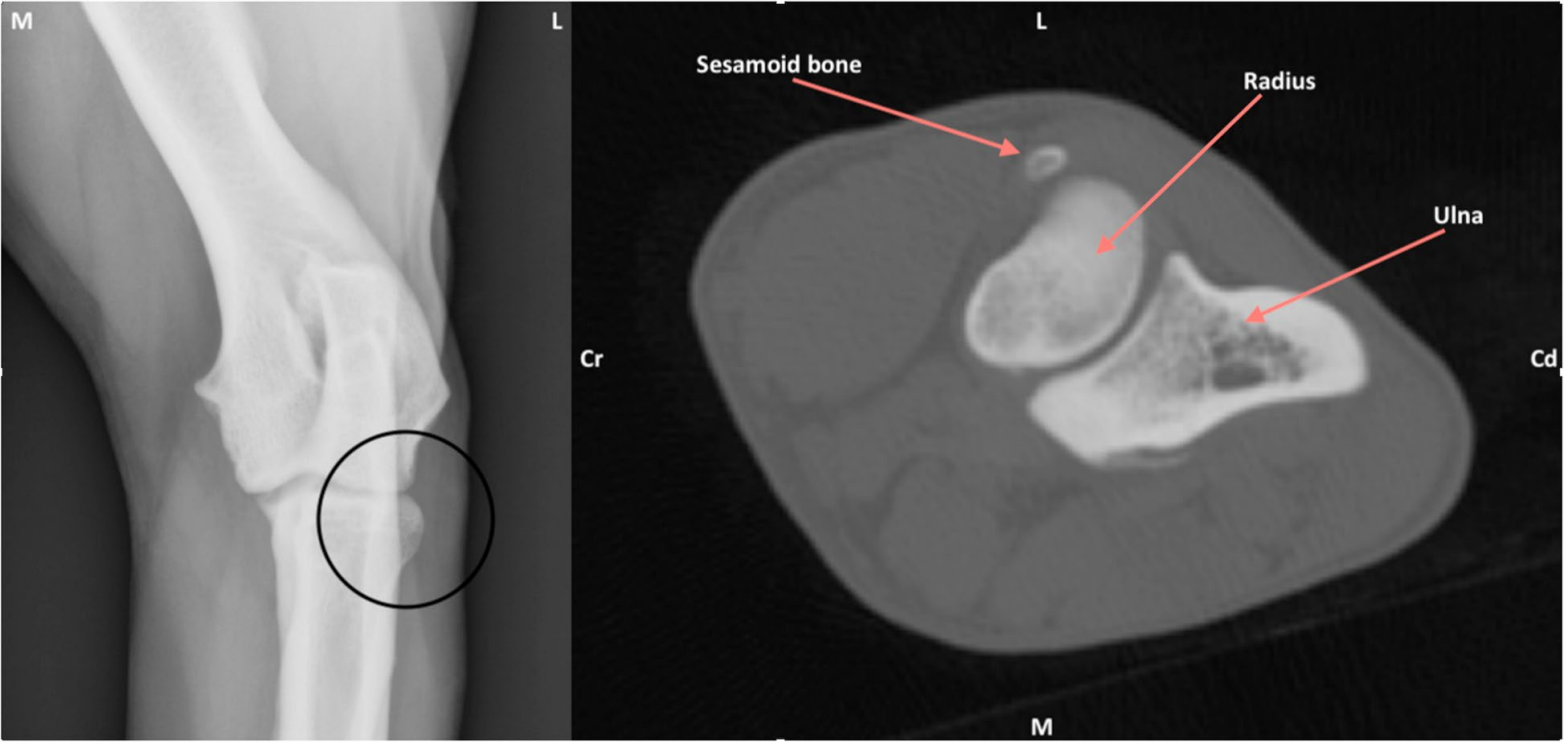
The craniolateral-caudomedial oblique (Cr15°LCdMO) (left) and transverse CT image (right) of the right elbow of the same dog. There is no sesamoid bone detectable on the radiograph craniolateral of the head of the radius (black circle), however the sesamoid bone can be detected clearly on the CT image (marked with arrow)

**Fig. 2:**
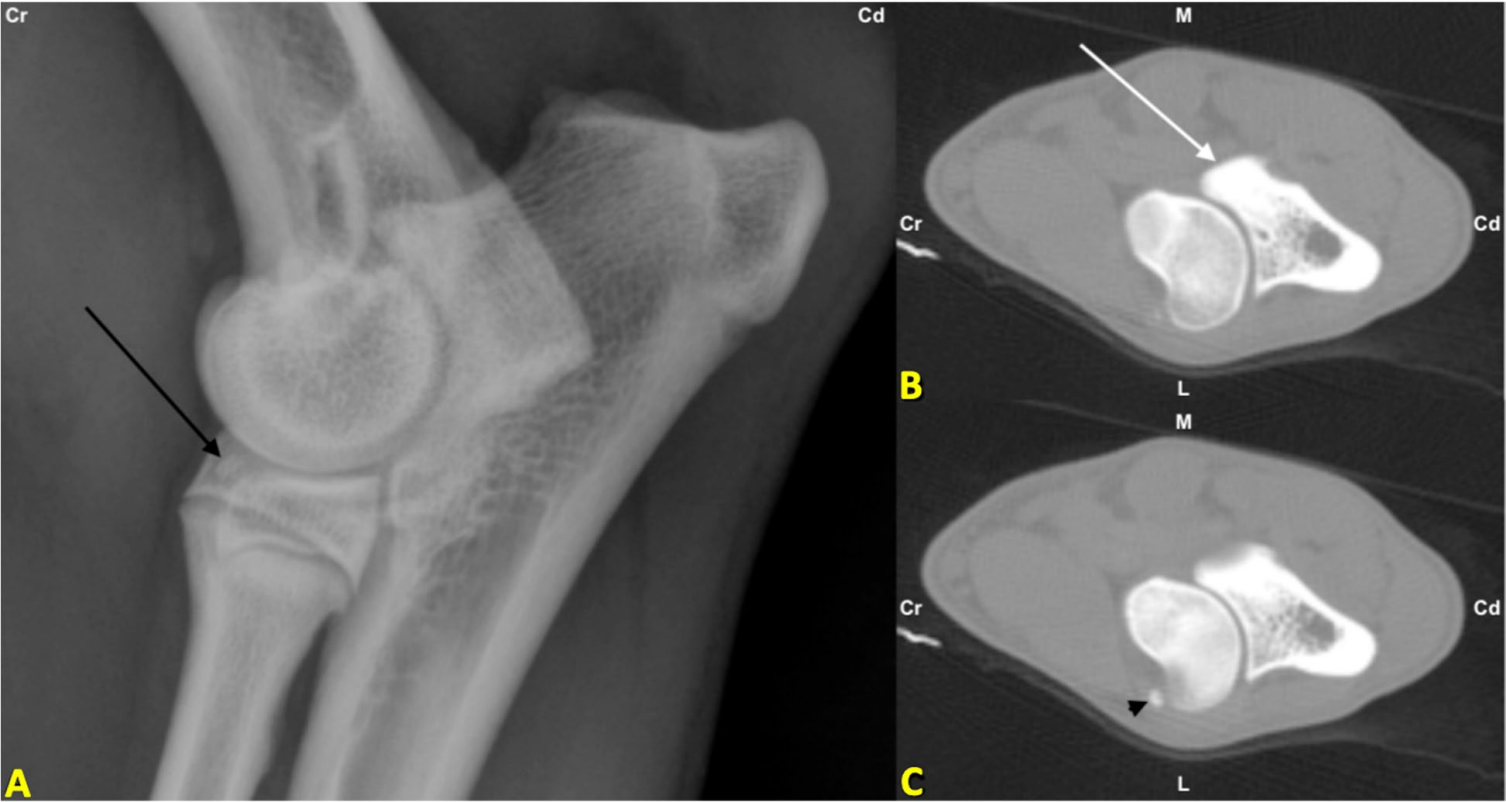
**A** Lateromedial radiograph of the left elbow. The black arrow marks a bony opacity; **B** CT scan of left elbow of same dog. The white arrow marks the intact medial coronoid process; **C** CT scan of the left elbow of the same dog. The black arrowhead marks the sesamoid bone in the supinator muscle

The average age of the dogs with detectable SSBs was 14 months. On radiography, a SSB was detected unilaterally in 44.44, 62.5 and 62.5% of dogs and bilaterally in 55.56, 37.5 and 37.5% by observer 1, observer 2 and observer 3 respectively (Table 1). On CT, all 3 observers identified a sesamoid bone in 26% of the dogs, and 76.92% of these dogs had bilateral SSBs. An average of 72% of the sesamoid bones were not identified on radiography but were detected in CT scans (Fig. 1). An average of 28% of the SBBs was detected in both radiographs and CT scans (Fig. 2). The detected SSBs were round to oval-shaped measured 0.5–6.56 mm × 0.5–6.2 mm in radiography and CT examinations. On both X-ray and CT scans the SSBs were detected as uniform mineral opacities with smooth well-defined borders. Additionally, the medulla and cortex of the SSBs were visible on CT images.

**Table 1:** Detected supinator sesamoid bones by 3 observers in radiographs and CT images

Detection of SBB	Obs. 1	Obs. 2	Obs. 3	Average
Total dogs with sesamoid on X-ray (100 dogs); in these dogs:	9%	8%	8%	8.33%
- unilateral	44.44%	62.5%	62.5%	56.48%
- bilateral	55.56%	37.5%	37.5%	43.52%
Total number of sesamoid bones on X-ray (200 elbows)	14/200 (7%)	13/200 (6.5%)	11/200 (5.5%)	12,67/200 (6.3%)
Total dogs with sesamoid on CT (100 dogs); in these dogs:	26%	26%	26%	26%
- unilateral	23.08%	23.08%	23.08%	23.08%
- bilateral	76.92%	76.92%	76.92%	76.92%
Total number of sesamoid bones on CT (200 elbows)	46/200 (23%)	46/200 (23%)	46/200 (23%)	46/200 (23%)
Missed sesamoids on X-ray	32/46 (70%)	33/46 (72%)	35/46 (76%)	33.33/46 (72%)

Based on the moderate agreement between the observers in the evaluation of the radiographs, an average of the numbers of the detected SSBs was calculated and reported. No average calculation was needed to present the CT scan’s results, as the 3 observers found the same numbers of SSBs. An average number of the detected SSBs unilaterally and bilaterally on radiographs was calculated; 8.33 and 43.52% respectively.

**Fig. 3:**
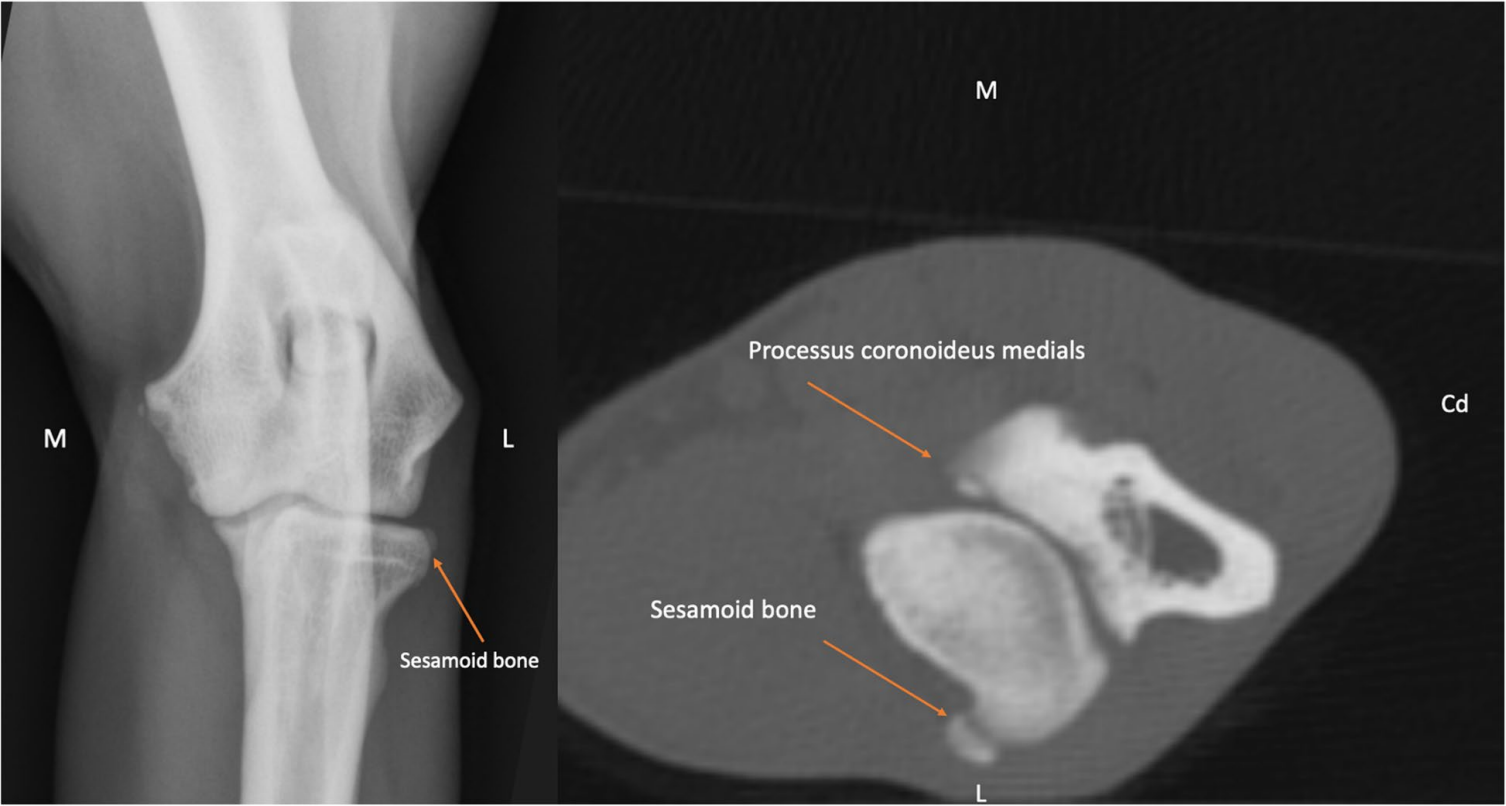
The radiograph (left) and transverse CT image (right) of the left elbow of the same dog. The sesamoid bone is detectable on the radiograph and CT scan (marked with arrow). A non-displaced fragment is detectable on medial coronoid process (Elbow dysplasia)

An average of 50% of the dogs had radiological evidence of an elbow disease in one or both elbows. Forty-one dogs had medial coronoid process disease, 21 dogs had osteochondrosis, 20 dogs had incongruity, 7 dogs had incomplete ossification of the humerus condyles and ununited anconeal process was detected in none of the patients (some dogs had more than one disease). An average of 29.67% of these affected elbows contained a SSB (Fig. 3). The statistical analysis revealed 44% chance of having elbow disease without a sesamoid bone and a 70% chance to have elbow disease with a sesamoid bone. Statistical analysis, using a chi-square test, showed that there was a significant correlation between elbow disease and the presence of a SSB (χ2 = 9.1, *p*-value = 0.002). The Phi and Cramer’s V values were 0.214.

## Discussion

In the present study, SSB was detected radiographically on average of 8.33% of the dogs; a bilateral sesamoid bone was observed in 43.52% of these dogs. On CT, a SSB was found in 26% of the dogs and in 76.92% of these dogs a bilateral sesamoid bone was observed. An average of 72% of the SSBs was missed on X-ray. The sesamoid bone was observed as round to oval-shaped with a diameter of 0.5–6.56 mm × 0.5–6.2 mm. The Kappa test showed a substantial agreement in the detection of SSBs in radiography and perfect agreement in CT. A weak positive association was detected between the existence of elbow diseases and SSBs.

Detection of the SSBs is not only important in diagnosing the elbow dysplasia- as they can be confused for a fragmented coronoid process in the lateral radiograph- but also important in the management of the lameness. It has been reported that surgical removal of SSB in eight lame dogs resulted in a good outcome [6]. However, the presence of a SSB causing or worsening lameness has not been proven. The description of the SSBs in the present study as a round to oval well-defined opacities with a diameter of 0.5–6.56 mm × 0.5–6.2 mm in both radiography and CT examination, which may be present unilaterally or bilaterally can help the observers to detect them.

Previous studies reported the presence of SSB in 9.4, 11 and 31% of their respective canine population by means of radiographic assessment [3, 7, 8]. The difference between the results of the latter study [3] and the other studies [7, 8] was due to the different methodology. The supinator muscle was dissected and radiographed solely to avoid the superimposition [3] which resulted in detection of a higher number of SSBs. In the present study, the superimposition was avoided by using CT and our hypothesis that a higher number of sesamoid bones would be detected on CT scans was confirmed. In this paper, a SSB was detected in 26% of the dogs by means of CT imaging which is closer to the percentage presented by Wood et al. (1985) [3]. The lower percentage of the detectability of the SSBs in radiography with standard positioning is therefore considered primarily due to superimposition of overlying structures on radiographs. Methods used to avoid superimposition, such as radiography in different projections and CT scanning, will increase the detectability of SSBs.

The SSB is located craniolateral to the head of the radius. To properly detect the sesamoid bone the radiographs should be taken in a craniomedial to caudolateral oblique projection, as this view reduces superposition of the radial head on the sesamoid bone [9]. However, this view is not included routinely in the standard radiographic survey of the elbow of dogs. Flückiger (2010) advised to pronate the elbow 15° to take the craniocaudal radiograph [10], as by pronating the elbow a craniolateral-caudomedial oblique (Cr15°LCdMO) view can be taken without changing the position of the X-ray tube [11]. On the pronated craniocaudal radiograph the sesamoid bone of the supinator muscle is often superposed on the radial head and therefore difficult to identify. On CT images there is no superposition, which makes it easier to visualize the sesamoid bone.

There are two theories to explain the existence of the SSBs. Generally, all the sesamoid bones are physiological parts of the musculoskeletal system. However, Wood et al. (1995) [2] and Meyer-Lindenberg et al. (2004) [12] suggested that sesamoid bones may form in response to repeated stress and excessive mechanical friction occurring in a tendon or joint capsules and that their important role is to protect the tendon from the adjacent boney structures in physiological or pathological conditions and change the direction of the stress. On the other hand, other studies regard the sesamoid bone as a vestigial structure and consider it to be a phylogenetical remnant of a primitive supplementary bone [13].

Based on the first theory, it is logical that any disorder which result in a change of the weight bearing and consequently change the stress in the elbow joint may provoke the development of the SSBs. It has been described that an altered mechanical situation in elbow diseases will result in a weight bearing change and a load-transfer shift [14–16]. Ariffin et al. in 2015 [4] demonstrated that the presence of a radiographically visible SSB is related to cartilage pathology and that its presence can be an indicator of osteoarthrosis. The present study revealed a positive correlation between elbow disease and presence of a sesamoid bone in the supinator muscle.

Histopathologically, the tendon of the supinator muscle often includes areas of chondro-osseous metaplasia in some species [17], so it would be possible that an abnormal gait and consequently increased stress on the tendon of the supinator muscle causes a fibrocartilaginous metaplasia and formation of the sesamoid bone to support the tendon [2]. Alternatively, it could be due to the mineralization of the prenatally existed cartilaginous SSBs during arthritic changes that then becomes radiographically visible [4]. It is important to note that the presented correlation doesn’t necessarily means that there is a causation as other variables possibly influence this matter. It was also not taken into account that two sesamoid bones could be from the same dog. The statistical test was performed on individual elbows and not per dog. Since most dogs have bilateral sesamoid bones, this can also influence the statistical results.

Ariffin (2015) [4] described the detection of dense regular connective tissue or non-mineralized cartilaginous cap in the cases with no radiographically detectable supinator sesamoid bone in cats. Accordingly, the main limitation of the present study is the lack of necropsy and histopathology to check the existence of non-mineralized sesamoids due to the in vivo nature of the study. Additionally, due to the retrospective nature of the study and based on the radiation safety regulations, a serial radiographic assessment or a control CT examination were not available to evaluate the development of SSBs and ED chronologically. Furthermore, only radiographs with standard positioning were available and evaluated in the present study which may have influenced the visualization of the SSBs in the radiographs. However, the closure of proximal radial growth plate may affect the detectability of the SBBs due to a superimposition, an evaluation of this effect was limited in the present study due to a lack of defined age groups. Additionally, based on the retrospective design of the study, the male and female dogs were included unequally in the present study. Consequently, effects of the sex could not be evaluated on identification of SSBs without bias. A prospective or experimental study would be needed to overcome these limitations and permits further evaluation of the SSBs.

In conclusion, the SSBs were less frequently detected in radiography of the canine elbow joints when compared with CT examinations with a substantial interobserver agreement. Due to superimposition of the overlying structures, CT examination can be considered as a method of choice to detect SSBs with almost perfect interobserver agreement. The presented correlation between the existence of the SSBs and elbow diseases, potentially raises the practical concern in elbow dysplasia screening, thus, further cohort study is recommended to follow this correlation.

## Data Availability

The datasets used and/or analysed during the current study available from the corresponding author on reasonable request

## Abbreviations

CT: Computed Tomography
Cr15°LCdMO: 15° Craniolateral-Caudpmedial Oblique
ED: Elbow Dysplasia
SSB: Supinator Sesamoid Bone
